# Out with the new, in with the old: how traditional masculine norms are constructed, communicated, and contested within an all-boys’ school community

**DOI:** 10.1080/00049530.2026.2679704

**Published:** 2026-06-10

**Authors:** Auguste G. Harrington, Sarah Moss-Holland, Jacob Kuek, Ashley Neilson, Amany Gouda-Vossos, Khandis Blake

**Affiliations:** a Department is Psychology, New York University, Abu Dhabi, United Arab Emirates; b Melbourne School of Psychological Sciences, University of Melbourne, Parkville, Australia; c Research Computing Services, University of Melbourne, Parkville, Australia

**Keywords:** Masculinity, adolescence, traditional masculine norms, socialization

## Abstract

**Objective:**

Previous research has explored adolescent boys’ masculinity attitudes and the social forces shaping them. We extend this work by examining masculinity attitudes of all students, parents, and staff of a relatively affluent, independent faith-based all-boys’ school in metropolitan Australia.

**Method:**

A qualitative Reflexive Thematic Analysis of open-text questions, distributed in an online survey to 650 students, 287 parents, and 46 staff, examined beliefs about masculinity, desired masculine ideals, contextual differences (home vs. school), and behavioural consequences.

**Results:**

Boys’ accounts revealed context-dependent constructions of masculinity, with pro-social norms more salient at home and dominance-oriented norms enforced in school settings. They framed traditional masculinity as developmentally necessary, reinforced by manosphere narratives and fuelled by counter-cultural resistance to progressive change. Despite this, many boys recognised the personal and social costs of these norms, and there was substantial divergence between boys’ views and those of parents and school staff.

**Conclusions:**

Results highlight the influential role of peer/school contexts and online manosphere narratives in shaping Australian boys’ masculinity attitudes, as well as clear generational differences in how masculine status was defined. These findings point to schools as key leverage points for interventions aimed at promoting healthier, context-sensitive models of masculinity.

## KEY POINTS

**What is already known about this topic:**

1. Endorsing traditional conceptions of how men should think, feel, and behave is associated with negative outcomes for men, boys, and the people around them.
2. Adolescence is a critical period for the development of these attitudes.
3. Peers, parents, and teachers likely influence boys’ attitudes about masculinity.

**What this topic adds:**

1. Australian boys’ masculinity attitudes were highly context-dependent, with more pro-social ideals expressed at home and dominance-oriented norms most strongly reinforced in school settings.
2. Peer cultures and online manosphere narratives emerged as particularly influential, often outweighing parental and institutional messages about masculinity.
3. Substantial misalignment across generations was observed, with boys’ conceptions of masculinity often diverging from those of parents and school staff, highlighting limits of adult-led approaches to shaping boys’ masculinity.

Across the world and the lifespan, social pressures and expectations motivate and constrain men and boys into possessing “traditional” masculine traits, such as power, agency, and independence, and avoid feminine traits such as communality, deference, and nurturance (see Kågesten et al., 2016 for review). Greater belief in the importance of traditional masculine norms is associated with a range of negative outcomes for men, boys, and the people around them, including emotional suppression, aggression, and risk taking (see Levant & Richmond, 2007 for review). Consequently, it is important to understand the content and sources of these attitudes.

Adolescence has been identified as a developmental period central to the acquisition of gendered attitudes (see Halimi et al., 2016; Kågesten et al., 2016; Rogers et al., 2021 for reviews). During this period, social (e.g., increased importance of peer acceptance; Smetana et al., 2016), psychological (e.g., increasing cognitive capacity for abstract and social comparison; Leman & Tenenbaum, 2017), and physiological (e.g., puberty, the prominence of male sex characteristics; Hill & Lynch, 1983) transitions shape attitudes and beliefs about masculinity. Prior scholarship indicates that the content of adolescent boys’ masculinity attitudes remains remarkably traditional, that is, hegemonic, white, western, wealthy and cis-gendered (Kågesten et al., 2016; Pleck, 1995). Adolescent boys continue to feel pressure to adhere to traditional masculine norms, and report adherence to, and endorsement of, these norms around the world (Kågesten et al., 2016).

A range of research has explored the sources of boys’ masculinity attitudes, broadly indicating that parents, teachers, and peers all play a role in the socialization process (Halimi et al., 2016; Kågesten et al., 2016). For instance, parents’ attitudes tend to be congruent with those of their children (e.g., Degner & Dalege, 2013), and this influence emerges through both direct (i.e., verbal messages; Epstein & Ward, 2011) and indirect channels (e.g., differential treatment by gender; Berke et al., 2018). Likewise, teachers perpetuate traditional masculine norms by modifying the type (e.g., authoritarian vs comradely) and valence (praise and chitchat vs scorn and antagonism) of attention directed towards students, preferencing those who conform to these norms (e.g., Smith, 2007; Tischler & McCaughtry, 2011). Finally, there is particularly strong evidence for the influence of same-sex peers on the socialization of gendered attitudes during adolescence (Kågesten et al., 2016; Rogers et al., 2021). Social punishments and rewards are used by boys’ peers to “police” gender conformity, with masculine-norm conforming boys receiving social acceptance and status, while non-conforming boys experience ostracism, ridicule, and other forms of bullying (Reigeluth & Addis, 2016). Boys report consciously adapting to these pressures to secure their position in their peer groups (Reigeluth & Addis, 2016).

Importantly, the socialization of gendered attitudes does not occur in a vacuum, and context has long been recognised as influencing this process (e.g., Deux & Major, 1987). Most boys’ lives revolve around two primary social contexts: home and school. There is little research assessing the influence of home context on boys’ attitudes beyond their socialization by parents. More attention has focused on the context of school, indicating that schools are central to the development and maintenance of traditional masculine attitudes (Halimi et al., 2016; Kågesten et al., 2016). All-boys’ schools in particular have long been theorized to be a context in which traditional masculinities flourish (Connell, 1996). Indeed, empirical work suggests that all-boys’ schools uphold traditional masculine norms (and encourage adherence to these norms) through institutional prescriptions, proscriptions, traditions, and status hierarchies that reward traditionally masculine boys (e.g., Howard & Keddie, 2023; Tischler & McCaughtry, 2011).

Another increasingly important social context in boys’ lives is the digital environment. Digital spaces provide a vast wealth of information and potential peers for boys to engage with (Pasquier, 2008). Some of this information is alternative and contrary, countering what they are taught at home and school. The vastness and contrariness of information in the digital space can be a source of appeal for boys: Adolescence is characterised by differentiation, especially from parents, and engaging with alternative information and holding contrary views can assist boys in forming their own identity (Erikson, 1986).

The potentially potent socialization force of digital spaces has led to growing concern among parents, educators, and policy-makers for the online “manosphere”. The manosphere is a loose network of communities that claim to address men’s struggles, but mostly promote a version of masculinity that is traditional, dominance-focused, and misogynistic (Ging, 2019). Manosphere groups and influencers are united by an opposition to women’s rights and a view that men are victims of the current social and political climate (United Nations, 2024). Manosphere content is increasingly being accessed by young men, with roughly two-thirds of young men across Australia, UK, and USA engaging with masculinity influencers online (Movember, 2025). This increased access is concerning not only because manosphere ideology is harmful to men, boys, women, and girls, but also it has growing links to violent extremism (Meger et al., 2024).

### Current research

While a range of previous research has explored the content and sources of boys’ attitudes about masculinity, these previous studies share a key limitation: Namely, they examine boys’ attitudes about masculinity through isolated snapshots of boys’ social lives. For example, some studies have explored the content of boys’ attitudes, without consideration of how social contexts like home versus school, and the shared meanings constructed in these spaces, differentially shape these attitudes (e.g., Howard & Keddie, 2023; McNulty & Birney, 2023). Others have examined whether boys share attitudes with their parents, without accounting for other social figures like teachers or online influencers (e.g., Epstein & Ward, 2011). Although all such studies provide invaluable contributions, understanding how boys’ masculinity attitudes are constructed, conceptualized, and endorsed at home and school, as well as differences between students, parents, and teachers across these contexts would be a useful extension of prior knowledge. To contribute to this aim, we will conduct a Reflexive Thematic Analysis on a large qualitative dataset encompassing open text responses from all participating students, parents, and staff of an all-boys’ school in Australia. This approach allows us to simultaneously explore 1) how boys experience, construct, express, and engage with masculinity, 2) similarity between boys’ attitudes and those of their peers, teachers, and parents, and 3) how these attitudes and beliefs differ across social contexts.

## Methods

Participants were recruited from Briarwood College (pseudonym), a large, faith-based independent boys’ secondary school located in metropolitan Australia. The school enrols over 1000 students across Years 7–12 (ages 11–18) and serves a predominantly socioeconomically advantaged, though not elite, population, with many families reflecting upward socioeconomic mobility and backgrounds more characteristic of skilled trades, small business ownership, and mixed white- and blue-collar employment. The student body is largely of European descent. The school also has a strong sporting culture and functions as a recognised pathway for competitive school and representative sport, a feature that may further shape local norms surrounding masculinity, status, and peer recognition.

Approval was obtained from the University of Melbourne Office of Research Ethics and Integrity prior to the start of data collection (Reference ID #: 2022-25570). Data were collected in a de-identified manner and pseudonyms used throughout. At no point did the author who chose the pseudonyms have access to the names of participants in this study and thus selected the pseudonyms entirely at random. Any resemblance between pseudonyms used and any participant’s real name is purely coincidental. All staff, students, and parents were invited to participate in a study on “the culture of masculinity at Briarwood College”.

Students completed surveys online, privately and silently in the classroom. Boys were instructed to participate in the online survey if they wanted to, and if they didn’t want to, they completed homework independently and silently. There was no penalty for non-participation and participation was anonymous: Teachers were explicitly instructed not to monitor or encourage participation, other than instructing boys in how to access the survey link and ensuring they were engaging in their work independently and without affecting other students. Parents and staff also completed the survey in their own time. Although the level of engagement with the survey questions varied, most students, and the majority of parents and staff, provided detailed answers indicating a high degree of engagement with the questions. Younger students were relatively more likely than older students to provide shorter responses (e.g., one-word answers).

The current research focused on qualitative data collected as part of a broader study in which participants completed: 1) ten qualitative questions, 2) quantitative questions intended to inform future intervention efforts, and 3) limited demographics. 1,313 people consented to participate, but 329 people did not answer any qualitative questions and 10 responses were unintelligible. All participants who complete the study were entered into a raffle to win one of 5 × $50 gift cards. Our final sample included 986 participants, including 650 students, 287 parents, and 46 staff, and demographics are in Table 1. The qualitative survey questions were developed by the research team and subsequently approved by an advisory group within Briarwood College. They were designed to elicit detailed self-reflective response regarding: 1) beliefs and conceptions about masculinity across various domains, 2) desires for what masculinity should be, 3) perceptions of differences in masculinity across contexts, and 4) how masculinity influences behaviour (see Appendix Section 1).

**Table 1.** Demographics of the sample employed in analyses.

Demographic	*n* (%)	*M* (*SD*)
Age		
Students		14.80 (1.75)
Parents		49.55 (5.08)
Staff		44.58 (10.57)
Gender		
Students		
Male	632 (97.2)	
Other	18 (2.77)	
Parents		
Male	93 (32.2)	
Female	194 (67.1)	
Other	0 (0)	
Staff		
Male	12 (26.1)	
Female	34 (73.9)	
Other	0 (0)	
Ethnicity		
Europe	576 (58.42)	
Australian/NZ	526 (53.35)	
Asia, South-Eastern	40 (4.06)	
Africa	32 (3.25)	
Indigenous Australian or Torres Strait Islander	34 (3.45)	
Asia, Southern	31 (3.14)	
Asia, Western	24 (2.43)	
Asia, Eastern	15 (1.52)	
North America	15 (1.52)	
South America	12 (1.22)	
Polynesia	6 (0.61)	
Central America	6 (0.61)	
Latin America and the Caribbean	6 (0.61)	
Asia, Central	3 (0.3)	

*Note*. *N* = 986. Participants could select more than one ethnicity and thus percentages sum to greater than 100%. Although 18 students selected their gender as “other”, when asked to describe their gender identity, all did so in a way that suggested their intention was to satirize and/or derogate non-binary gender identities (e.g., “I am a two-sprit transgender nonbinary fish”, “attack helicopter”, “tv remote”), thus we caution against interpreting the gender identity of these participants and refer to all students in the sample as “boys” for concision.

### Researcher characteristics and reflexivity

Our research team included members with a diverse range of expertise, lived experiences, and perspectives. Three of the research team identified as women, and three identified as men. As our goal was to leverage this diversity to more effectively explore our research questions, we undertook a reflective exercise before engaging in analysis. The exercise revealed that members had varied professional and personal backgrounds, including secondary and tertiary education and interdisciplinary research (psychology, biology, data analytics, sociology). Members’ knowledge about masculinities scholarship differed, but all had professional and/or lived experience with masculinities and were positively oriented towards utilizing qualitative methodology and committed to research openness and rigour. Due to this diversity of knowledge and beliefs, expectations about what the data would demonstrate were informed by both 1) relevant prior research related to masculinities within and outside of educational contexts (e.g., Halimi et al., 2016; Kågesten et al., 2016; Rogers et al., 2021) as well as 2) our individual lived experiences interacting with men and masculinities as men and women. With this understanding – and an acknowledgement of our individual subjective positions – we aimed to leverage our diversity of perspectives and backgrounds through a reflexive approach to analysis. Namely, we aimed to enrich the research process by ensuring a multi-faceted understanding of masculinity within a community centred around an Australian all-boys’ school.

### Analytical approach

We employed Reflexive Thematic Analysis (Reflexive TA) to analyse qualitative written responses (e.g., Braun & Clarke, 2021). A constructivist paradigm informed the research design, recognising that students construct meaning of concepts based on their own beliefs and experiences, and through interactions with peers, parents, and educators (Creswell, 2017). Reflexive TA was chosen for its flexibility and alignment with constructivist principles, particularly its emphasis on the active role of the researcher in generating meaning from the data. Throughout the analytic process, reflexivity was systematically employed. Reflexivity uncovered varied ontological and epistemological views within the research team, including both positivist and postpositivist perspectives. To balance these varied ontological and epistemological views, we adopted a constructivist/interpretivist epistemology and a relativist ontology. Thus, we balanced our role as objective researchers aiming to further knowledge, with the postpositivist view that individuals’ (ourselves included) construction of knowledge and realities are dependent on context, culture, perspectives, and lived experience. We approached our subjectivities as a resource, elucidated through reflexivity.

### Analyses

Our Reflexive TA was conducted in NVivo. Analyses followed the six-stage process outlined by Braun and Clarke (2022), in a manner that fostered reflexivity. First, we familiarized ourselves with the data by reading through responses and reflecting on common trends. Second, we organized responses into codes based on reoccurring trends in the data, which were iteratively refined as familiarity with the data increased to strengthen the accuracy of representation. Third, we organized codes into themes (i.e., patterns of codes sharing a common idea). Informed by our relativist/constructivist/interpretivist approach, we balanced inductive and deductive reasoning when generating themes, drawing on common elements identified in the literature as well as leveraging our interpretation of the data to inform themes. Themes were then finalized, defined, and named during a meeting of the entire research team. Analytic sufficiency was determined through iterative engagement with the dataset, reflexive analytic memos (i.e., checking in on each other’s thought processes), and repeated review of theme coherence and distinctiveness. Analysis continued until themes were well-developed, conceptually meaningful, and capable of accounting for patterned variation across the dataset, rather than until no new codes could be identified.

## Results

Reflexive thematic analysis of participant responses identified five overarching themes: 1) Biological essentialism, 2) Traditional Masculinity, 3) Generational Masculinity, 4) Masculinity as a Subjective Construct, and 5) Rejection of Traditional Masculinity (see Table 2). The Traditional Masculinity theme included three subthemes 2.1) Toughness, 2.2) Status, and 2.3) Agency. These three subthemes, in turn, included nine further subthemes (Table 2).

**Table 2.** Themes extracted from qualitative analyses.

Theme	Definition	Sample Quotation
1. Biological Essentialism	Masculinity via male sex and physical characteristics of the male sex.	*“ … a male is not someone who identifies as a male, but is genetically a male. My mother cannot identify as a male and therefore is not my father. The characteristics of a male do vary from individual but essentially a male for me is genetics”.*
2. Traditional Masculinity		
2.1. Toughness	The need to exhibit mental and physical fortitude	*“I always try to look tough in front of my dad and my grandpa because they are two strong men and I really want to be like them […] so they don’t think less of me”.*
2.1.1. Strength	Possession and demonstration of physical and emotional strength	*“Masculinity is being a man which [sic] others perceive as being strong[…] showing no emotion, not being able to have sympathy […] for others”*
2.1.2. Irreverence	Mocking and/or unrefined behaviour	*“As a Briarwood student, masculinity looks like a bogan/uncouth behavior[sic] that is adopted, by a large majority of the boys at Briarwood. As many start swearing and use profanity to fit in, perform and act in outlandish/dumb ways, with myself being no exception”.*
2.1.3 Athleticism	Masculinity as defined by sporting prowess	*“[Masculinity is] to exhibit physical strength and athleticism[…]”*
2.2. Status	The achievement, possession, and demonstration of social status	*”[masculinity is] having to be better than everyone else”*
2.2.1. Respect	Attaining positive recognition within social groups	*“Masculinity needs to show respect and to be respected as it should inspire others to be confident, strong and a role model without abusing this trait”.*
2.2.2. Carrying out Male Responsibilities	Obligations to fulfil duties because of being a man	*“At home [masculinity] is to provide financial support for the family, take charge of household repairs and maintenance, and exhibit a stoic and authoritative demeanor[sic]”.*
2.2.3. Masculine Comradery	Masculine interdependence and masculinity as a boys’ club	*“I try to be more masculine at school when I am with my mates. We might not talk that much about feelings but [we] have mateship”.*
2.2.4. Bullying	Discrimination against those who are not conventionally masculine	*“There is bullying towards those who are different, don’t fit into the traditional sporting boxes. This is an ongoing issue […]”.*
2.3. Agency	The ability to exert and assert agentic control	*“A masculine man is one who is disciplined, has willpower to do things he doesn’t want to […]”.*
2.3.1. Strong-mindedness	Individual achievement through hard work and discipline	*“Excellence is not a destination; it is a continuous journey that never ends. Constant improvement is what fuels me, the endless objective to be better than who I was yesterday”.*
2.3.2. Independence	The absence of reliance on others	*“Masculinity should look like someone who can manage themselves especially when things get tough with lots of work”.*
3. Generational Masculinity	Masculinity changing across generations	*“Definitions of masculinity have changed since I was a boy. Now there is a full spectrum of masculinity whilst when I grew up there was a relatively narrow band”.*
4. Masculinity as a Subjective Construct	Masculinity as fluid and malleable across contexts and individuals	*“Every person has a unique view of what it means to be ‘masculine’ or a ‘man’. Masculinity is a subjective term that cannot be objectively defined […]”.*
5. Rejection of Traditional Masculinity	Explicitly or implicitly challenging traditional masculine norms	*“I try to instil a value system that is genderless and focuses on equity, respect and acceptance between men and women[…]”*

Together, these themes capture many distinct and often mutually-exclusive ideas articulated by participants, including both traditional, normative conceptions of masculinity (Themes 1 and 2), as well as more counter-stereotypical and nuanced conceptions (Themes 3–5). Despite the varied – and often seemingly contradictory – nature of participants’ ideas, themes and sub-themes were frequently articulated in intertwining ways. For instance, some boys simultaneously endorsed the benefits of traditional conceptions of masculinity, while also freely admitting the costs of such restrictive ideas – an apparent contradiction that we explore below. Some themes were also mentioned more frequently by parents and staff (Theme 1, Themes 3–5) than by boys (Theme 2).

In the following sections, we deconstruct these results using quotations reflecting patterns observed in the data – quotations selected for their clarity in articulating and illustrating patterns in the data. We first focus on student responses to understand how boys experience, construct, express, and engage with masculinity. We then draw on responses from boys, parents, and teachers to explore similarities and differences in their conceptions of masculinity. Finally, we consider how boys’ attitudes, beliefs, and conceptions of masculinity differ across the home and school contexts. Some quotations are particularly intense in tone and/or content, but differed from other more muted, subtle sentiments in degree, rather than in kind. High-intensity or sensationalized responses were not afforded greater interpretive weight during analyses.

### Boys’ conceptions of masculinity

Biological determinism of manhood/masculinity was present in boys’ responses, including rejection of, and bigotry towards, those identifying as male but deemed not to possess requisite biological characteristics (Theme 1). As with all subsequent themes, these patterns were evident across a wide range of responses, including those expressed in more moderate terms.

in my opinion masculinity defines the main biological and typical characteristics of a Male for example, high levels of testosterone, different hobbies than females, facial hair etcetera. – Ben, 13

Masculinity at Briarwood is beards and hairy men, Briarwood has heaps of them … the ideal man is not a trans and works hard to provide for his family and wife. He also dedicates his time to God. – Greg, 12

Boys’ conceptions of masculinity were closely aligned with traditional masculinity (Theme 2) across responses. Often, conceptions of masculinity centred around subjectively pro-social facets of masculinity. For example, manhood and high social status (Sub-theme 2.2) were consistently framed as achieved via earning and demonstrating respect, such as through carrying out male responsibilities, including doing good deeds, being kind, and generally possessing an unimpeachable moral standing. Likewise, boys frequently described manhood as defined by the embodiment of toughness and strength and resilience leveraged to protect and defend loved ones as well as those in need.

I believe that more traditional gender roles should be followed – Saul, 16

A man who is truly masculine embraces responsibility and loves, honours, protects and provides for his family and loved ones. He lives with integrity, motivated by conviction, not comfort or convenience. True masculinity is not determined by how much physical strength a man has but rather the strength of his character. – Jack, 17

However, more overtly anti-social masculine norms were strongly and consistently featured, including dominance, aggression, irreverent crassness, bullying, and bigotry.

[Masculinity at Briarwood] should look like a fit MAN asserting his dominance against the cowardly e.g.; overweight, unnecessary genders and sexualities. [Masculinity] feels like being masculine and have a great, fit, muscly body. [Masculinity] sounds like the man sticking up for himself and asserting his dominance. – James, 14

Concerningly, explicit and implicit references to “manosphere” masculinities—i.e., traditional, dominance-focused, misogynistic, and perpetuated by online influencers and groups (Ging, 2019) – commonly emerged in boys’ responses.

[Andew Tate’s] message is misunderstood. He is in jail because he teaches men to be strong, hold themselves accountable to their actions and to stop wasting their life away, or they will become irrelevant and pointless in this world. He believes a mans job is to be a provider to women and to have a happy life with traditional gender roles. Now this is not a bad thing at all, and I cannot stand people judge me for supporting him, when they do not consider the validity of their sources. – Nate, 18

Consistent with manosphere narratives, some boys extolled the benefits of traditional masculine norms in juxtaposition to “woke” (Kieran, no age provided) progressive attitudes, that they viewed as undermining social cohesion and stability. These benefits included encouraging boys and men to be agentic (Sub-theme 2.3), strong-minded and independent as well as athletic (Sub-theme 2.1). Some boys framed men as an “oppressed” group (Nolan, 17) and traditional masculine roles as devalued or marginalized by modern society.

i think the thought of masculinity scares people. [We are] told that being a masculine man these days is apparently misogyny […] men are currently being oppressed in todays society and you can [say] oh no no they’re definitely not what about the pay gap and the opportunity’s and the sports, almost all of them have been disproven, men no longer have the upper hand in society you do one thing wrong and your[sic] getting some sort of consequence where as women don’t get similar treatment in all cases […] i belive that in the community you get crucified for being a masculine man […] – Nolan, 17

Yet, many boys did not frame traditional masculine norms as uniformly positive nor without costs, instead highlighting the challenges and restrictions of living up to their prescriptions. For example, some boys reported believing that if they failed to live up masculine norms, both their loved ones and social reputation would suffer.

Our choices about being masculine can affect an entire comminity[sic]. Taking an initiative to do certain things can save people. But if a wrong choice is made it can be very financially damaging. – Will, 12

But as my dad struggles financially I don’t have the luxury of acting emotional and ‘getting in touch with my feelings – Charlie, 17

Likewise, boys consistently endorsed the importance of being respected, yet often suggested earning this respect requires invulnerability, infallibility, and imperviousness to hardship – expectations some felt to be a heavy burden. Many of the same boys who endorsed the importance of traditional masculinity, and/or lamented the marginalization of associated norms, subsequently indicated the pressure they felt to live up to these norms “no matter the cost” (Nolan, 17).

[At home, a masculine man should] take care of the people he loves and will protect them from everything no matter the cost at home this impacts me because i believe when something happens at home it is up to me to fix it from the simplest things to the worst of things – Nolan, 17

i want to be as strong as [my dad and my grandpa] and as masculine as them so they dont think less of me. – Bryce, 13

Some boys even explicitly identified the harmful consequences – both for themselves and others – of *successfully* adhering to traditional masculine norms, such as being motivated to behave in harmful, disrespectful, or disingenuous ways.

Sometimes [masculinity]can make me engage in poor choices – Mark, 17

[At school] I feel like I need to be dominant or competitive to be seen as masculine […] I need to be stoic and not show emotions [and] struggle to connect with others or to ask for help when I need it. – David, 17

However, many boys did not view anti-social and/or harmful aspects of traditional masculinity as wholly negative. For instance, some endorsed behaviours such as fighting, conflict, and bullying as an essential aspect of their development. Although these boys often recognized that such aggression may harm those unwilling or unable to conform to masculine norms, they portray this culture of conflict as the crucible that forges toughness, strength, and comradery between boys and facilitates their transition into manhood (Sub-themes 2.1 and 2.2). Thus, harms are framed as unfortunate by-products of an essential system.

[At Briarwood, masculine behaviour] causes bullying, discrimination, while also creating lifelong and strong brotherly bonds. Leaving some to thrive and others don’t. – Charlie, 17

The ideal man in the schoolyard is pretty rowdy and never flees from fighting as he knows that participating in brawls is a key to self development and the development of one’s courage. – Tim, 18

If boys set uncompassionate standards for others, they hold equally unforgiving personal standards, often taking an all-or-nothing perspective on what constitutes adherence to masculine norms. Most prominently, high achievement (e.g., financial success) and status (e.g., high power, dominance) are described as goals that *must* be achieved to earn masculinity, and thus respect (Sub-theme 2.2).

Elon musk and other successful men like Jeff Bezos or Andrew tate also show how success comes from working hard, they show how men shouldn’t be contempt[sic] with a mediocre life but should try their best to live the best possible life they can. – Derek, 17

Such sentiments often co-occur with the manosphere-linked ideas that, through toughness, hard work, personal sacrifice, determination, competitiveness, and an “alpha-male”/“sigma-male” (Rob, 16) mindset (i.e., a drive to dominate and out-compete others), any man can achieve wealth, power, and status.

[Andrew Tate, David Goggins, Tristan Tate] are my reason for why i am a beast. I assert all dominance across everywhere thanks to these guys. – Flynn, 15

[Masculinity is] Strong, not a bitch, not a soy boy, not a Briarwood College fag, thinking for myself, sigma male, Alpha male, domination, being an assertive male, who self improves. – Rob, 16

Consistent with these ideas, for many boys, their greatest concern was not the harms of traditional masculine norms, but rather that adherence to these may be put at risk by progressive change (Theme 3).

[I wish masculinity at Briarwood was …] being big and strong men who arent[sic] afraid to get their hands dirty and protect and provide for those they love. none of this acceptance in[sic] being weak – Philip, 17

I am happy with the masculinity that is currently going on at Briarwood. However, i wish that we don’t implement any views about identifying as something – Luke, 16

There was also variation and nuance in boys’ attitudes, with some boys describing masculinity as subjective, fluid, and malleable (Theme 4). These students suggested that masculinity is a dynamic concept which changes across settings, individuals, and backgrounds. Some students emphasised the detriment of holding a fixed version of masculinity, and recognised how fluid definitions can lead to inclusivity towards diverse masculinities. Yet these same ideas were drawn on by others as foolish, deluded, and contributing to societal ills.

I believe that masculinity is a spectrum, a person can be very masculine or not very masculine and some are somewhere in the middle, but they’re all considered masculine. - Praveen, 14.

Strict definitions can be harmful, perpetuate gender stereotypes, and limit individuals from expressing themselves authentically. Celebrating and acknowledging the diversity of masculinity allow[s] people to define it in their unique and meaningful ways. - Muhammad, 15

… In the greatest societies such as Bronze Age Greece, there was one definition of masculinity. Now in out [sic] demented and degenerated age, we believe masculinity to be more fluid, and the idea has only served to create a generation of aimless men who spend their days engaging in useless and self-damaging vice. - Tim, 18

There were also relatively isolated instances of boys’ rejection – implicit and explicit – of traditional masculine norms, such as expressing a desire for associated social pressures and expectations to be relaxed (Theme 5). Though, some boys did describe the “toxicity” of masculinity and negative outcomes as stemming from it.

I wish masculinity at Briarwood wasn’t toxic … – Isaiah, 14

[I wish masculinity at Briarwood was] a culture where boys and young men are encouraged to express themselves in ways that feel authentic to them, rather than feeling pressured to conform to narrow ideas about what it means to be a man. – David, 15

[I wish masculinity at Briarwood was] Kindness to everyone. Instead we have students talking how women deserve to have no rights and are inferior to “sigma males”. – Danny, 16

### Differences between students, parents, and staff

In this section, we examine differences in conceptions of masculinity among boys, parents, and staff. These comparisons emerged organically during analyses and thus reflect data-driven patterns of meaning, rather than formal comparisons based on a priori assumptions. More often than students, parents and staff (who were two-thirds female) expressed a fixed definition of masculinity based on biology (Theme 1), yet were similar to boys in many of their traditional beliefs about masculinity (Theme 2). For example, all three often endorsed the importance of men and boys possessing and demonstrating status, respect, and agency (Sub-themes 2.1 and 2.3.). However, parents and staff often offered a different, and notably more pro-social, interpretation of these constructs and how they should be achieved. Parents and staff conceived of status and respect as earned through prestige and high moral standing stemming from qualities like strong-mindedness, carrying out male responsibilities, and sometimes athleticism.

In the community, the ideal man contributes positively, engages in acts of kindness and service, promotes unity and harmony, respects diversity and inclusivity, and shows leadership in creating a better community for all. – Anthony, 64, father

In contrast, boys more frequently framed status and respect as won through qualities related to dominance, toughness, and independence.

Masculinity at Briarwood is being able to assert your dominance over the other kids to show that you are the man of the school and not to mess with them. – Bobby, 15

The divergences in attitudes between boys, parents, and staff are perhaps best illustrated by the not-uncommon framing of adherence to traditional masculine norms as a counter-culture rebellion. Boys described the virtue of adhering to masculine norms as an antidote to a societal trend towards less restrictive conceptions of men and masculinity (Theme 3). Moreover, they frame this trend as undermining men’s success and making them “soft, weak, and vulnerable” (Manny, 14). Boys frame those with less restrictive ideas (often parents and staff) as foolish, deluded, and contributing to societal ills.

… I believe that Masculinity is essential for a man and his purpose and my parents thinks[sic] its sexist and old fashion which I think is wrong. – Lawrence, 13

[My parents] see errors in my belief that men should aspire to reach there[sic] full potential, be physically and mentally strong, and be smart and focus on making money. I don’t see errors in this mindset. I do not wish to be a sheep. — Frank, 16

my family wants men to be the same to be soft weak and vunurlble[sic] where as we should be strong – Manny, 14

[I wish masculinity at Briarwood looked like] The teachers not teaching use[sic] woke principles and forcing information into our brains that aren’t nesseccary[sic] – Kieran, no age provided

Parents and boys were fairly balanced in their viewing of masculinity through a generation lens (Theme 3), although students’ references to this idea tended to focus more on what they perceived to be a modern degradation of masculinity. Parents’ and staff responses, on the other hand, tended towards encouraging a more fluid version of masculinity than students’ responses (Theme 4).

Why should young men compete to be the strongest, fastest or smartest? They are trapped inside of an imaginary pressure to conform to what a “man” should be. Who cares. Just be a good person. We need to make being a good person attractive. Not being a “masculine man”. – Kate, 31, Staff

Parents and staff also actively rejected traditional masculinity more often than boys (Theme 5). They described actively rejecting traditional norms by allocating household chores evenly to males and females, actively trying to instil emotional literacy in their sons, and promoting healthy masculine expression.

I have a partner who splits chores 50/50, understands I won’t take his name, […]. I also surround myself with males comfortable in expressing both societal “female” and “male” attributes … - Lisa, 33, staff

*I work hard to raise my two young men to be kind, helpful and to see that housework is the domain of both men and women in a household. […]. I encourage them to express how they feel and to talk through times of distress”. - Brenda, 56, mother*

As a male, I feel a responsibility to model what I believe are important masculine traits such as vulnerability and gentleness which are not considered part of masculinity … – Daren, 44, Staff

### Differences in masculinity between home and school

Boys’ responses consistently indicated that pressures and expectations they experience to embody masculine norms differ between home and school. Some boys described masculinity as less traditional and rigid – or even “non-existent” (George, 17) – at home compared to at school, and that this relative relaxation allows for greater freedom (Theme 4).

[At home] I make open choices that i may not make at school … i don’t really act brave or so masculin[sic] as i feel like there is no one around watching my every move. At home i can relax and be myself a bit more. – Terry, 14

At home masculinity is non-existent as everyone is treated fairly and can open up about anything. – George, 17

When masculine norms were described in the home context, they tended to centre around subjectively pro-social aspects of masculinity more often than at school. For instance, boys consistently endorsed the importance of fulfilling male responsibilities, including protecting and providing for loved ones – particularly women – by being responsible, respectable, and resilient (Sub-theme 2.2 and 2.3).

[Masculinity is] being the man of the house and showing that you are able to protect all the people in the house like my mum and my brothers. – Bobby, 15

Masculinity in my home is providing for the rest of the family. – Steven, 14

Being respectful, protecting our mums and sisters, being fair and just. Always being positive no matter the circumstance. – Herman, 16

Despite this comparatively pro-social climate, masculine norms present at home were not entirely free from restrictive prescriptions – although masculine norms at home were “not as pronounced” (Donny, 15) and more “relaxed” (Todd, 17) than those at school, they nonetheless came with an obligation to undertake difficult tasks, including “hard” (Donnny, 15) emotional and physical labour (Sub-themes 2.1, 2.3).

[Masculinity at home] looks like being strong and whilst I feel as though circumstances are relaxed at home after being at school all day there is still some expectation to act masculine whether it is doing a hard labour job every once in a while or killing a spider. [Masculinity] can also look like being emotionless even in the presence of your family. – Todd, 17

Masculinity in my home is not as pronounced as it is here at Briarwood, at home its peaceful. masculinity at home looks like the men and boys of the house doing the hard labour work, it also looks like working from 7am-7pm to provide for the family – Donny, 15

Moreover, boys’ “pro-social” conceptions of masculinity at home included a consistent thread of paternalism: Accounts often emphasized paternalistic expectations, including responsibility, emotional restraint, and provision-oriented roles (Sub-themes 2.1–2.3).

[An ideal …] A man goes to work and pays off the bills while his wife takes care of the kids. The mother of the kids turns the children into boys and then the Dad turns the boys into men – Daniel, 14

[Masculinity] At home is to provide financial support for the family, take charge of household repairs and maintenance, and exhibit a stoic and authoritative demeanor[sic], while expecting women and children to fulfill domestic and nurturing roles. – Jono, 15

Boys’ beliefs in the importance of protecting and providing for women appeared to be founded on gender hierarchies that place women in subjectively positive – but inherently limited and subordinate – roles in relation to men (sub-theme 2.2). In some responses, this “benevolent” sexism gave way to more overtly “hostile” sexism (see Glick & Fiske, 1997), including hints of the drive for dominance and control that frequently characterized conceptions of masculinity at school. Thus, `differences between home and school contexts appeared to reflect qualitatively distinct masculine norms, rather than a simple reduction in masculine restrictions.

[Masculinity at home is] Male figures being in control over the house and living space. – Norman, 14

[Masculinity at home is] Being the person who rules and is in charge of the house. And who provides the most to the family. – Elijah, no age provided

## Discussion

The current study explores how masculinity is conceptualized, constructed, and endorsed within a community centred around an Australian all-boys’ school. To do so, we examined attitudes about masculinity at home and school, as well as differences between students, parents, and school staff that emerged across these contexts. This approach allowed us to uncover the content of masculinity attitudes across the entire community, as well as the nuanced ways these attitudes are expressed, performed, and enforced. Our approach also provided a unique level of insight into possible sources of Briarwood boys’ attitudes: Boys’ attitudes more closely aligned with those of their peers, the manosphere, and the broader school context more so than their parents or school staff.

Overall, boys described masculinity as highly context dependent. In the home, masculinity was often articulated in pro-social terms and experienced as relatively less dominance-oriented – and even as a source of relief – although sometimes paternalistic and restrictive in other ways. In contrast, traditional masculinity was most strongly constructed and enforced in school settings, where anti-social behaviours such as aggression and bullying were frequently framed as necessary for development, group cohesion, and the transition to manhood. Some boys’ narratives aligned with manosphere-style conceptions of masculinity that emphasise dominance, status, and misogyny, often accompanied by portrayals of men as socially marginalised. Relatedly, adherence to traditional masculinity was sometimes framed as a counter-cultural response to “woke” progressivism, with greater concern expressed about preserving masculine norms than addressing their potential harms. These accounts stood in clear contrast to those of parents and school staff, who more often conceptualized masculine status as earned through moral standing and prestige rather than dominance. Many boys nevertheless demonstrated awareness of the personal and social costs of traditional masculine norms, acknowledging that adherence can motivate harmful or inauthentic behaviour despite perceived benefits.

### The contents and nuance of Briarwood boys’ attitudes about masculinity

At Briarwood, boys’ conceptions of masculinity appear to be strongly informed by traditional masculine norms, and boys endorsed these norms to a high degree, particularly within the school context. These findings align with previous research indicating that adolescent boys’ masculinity attitudes remain traditional (Kågesten et al., 2016), but contrast with work suggesting that increasing egalitarianism around the world is driven by less traditional attitudes within younger generations (e.g., Shu & Meagher, 2018).

Although appearing markedly traditional, boys’ masculinity attitudes were also complex, variable, and nuanced. For example, bullying emerged as a prominent strategy for policing adherence to masculinity, consistent with previous scholarship (e.g., Reigeluth & Addis, 2016). Yet, boys simultaneously lamented how pressures and expectations driven by masculine norms motivate harmful behaviours, including bullying. Additionally, many boys expressed relief at the relatively less dominance-oriented masculine norms characterizing the home context. Why then might boys endorse and conform to traditional masculine norms if they recognize – and even personally feel – their restrictions and harms? Our results provide a possible explanation: Boys described negative outcomes of masculine norms (such as bullying) as regrettable, but nonetheless essential, tools for facilitating character development, group cohesion, and the transition of boys into men. Thus, harms of traditional masculine norms are seen as having a net-positive effect.

However, the consistent framing of traditional masculine norms as essential and beneficial may not indicate straightforward internalization of these ideas. For instance, the expression of traditional attitudes could reflect a coping strategy whereby boys actively demonstrate, endorse, and perform what they perceive to be socially desirable attitudes and identities to gain social capital, despite not strongly internalizing these themselves. Nevertheless, such strategic performances serve to elucidate the culture of masculinity within the contexts they are deployed, shedding light on the normative constraints boys face. Moreover, repeated performances in line with traditional masculine norms may also contribute to their eventual internalization (Bem, 1967). From this perspective, boys’ narratives – whether grounded in internalization or social performance – provide evidence of dominant conceptions of masculinity within the school context and the socialization pressures boys face.

Thus far, we have treated “traditional” masculinity as a unitary construct encompassing restrictive, stereotypical conceptions of how men and boys should be. However, masculinities scholarship is heterogenous, encompassing a range of overlapping constructs, such as hegemonic masculinity (Connell, 2020), masculine role strain (Pleck, 1995), and ambivalent sexism (Glick & Fiske, 1997). These constructs differ in theoretical emphasis, yet share a common core: That masculinities motivate and constrain men into restrictive roles prescribing power, agency, and independence. Indeed, measures derived from these traditions are highly correlated (e.g., Krivoshchekov et al., 2023), illustrating that while scholars draw distinctions among these constructs, lay understandings do not necessarily reflect this nuance. Accordingly, our operationalization of traditional masculinity reflects both theoretical divergences across theories, as well as to their shared foundations – both in theory and in lay understandings. This approach aligns with our reflexive analytic strategy, integrating theory-driven interpretation with inductive attention to participants’ meaning-making.

### Socialization figures informing Briarwood boys’ attitudes about masculinity

Our sampling approach allowed for unique insight into the possible influences on Briarwood boys’ attitudes about masculinity. Boys’ responses suggest that social expectations and pressures from peers play a substantial role in shaping their attitudes, despite variation among boys in conceptualizations of masculinity. In contrast, boys’ reports suggest that parents and staff do not influence their attitudes to the same extent, and divergences emerging between the attitudes of these groups support this idea. These findings are consistent with research indicating a strong effect of peers on boys’ masculinity attitudes (Kågesten et al., 2016; Rogers et al., 2021), particularly within all-boys’ schools (e.g., Howard & Keddie, 2023), and that peers have a greater influence than parents on boys’ attitudes (e.g., Endendijk et al., 2022). However, these results contrast with scholarship demonstrating the strong influence of parents in adolescent gender socialization (Halimi et al., 2016), and teachers in gender socialization within all boys’ schools (e.g., Smith, 2007; Tischler & McCaughtry, 2011).

Despite the progressive attitudes characterizing staff responses, school context appeared to be an important influence on boys’ masculinity attitudes, as traditional masculine norms were reported as being performed, constructed, and enforced most often (although not exclusively) within the school context. In contrast, boys often described masculinity as being less restrictive or even “non-existent” at home. Yet, boys’ apparent belief in the benefits of masculine norms – often juxtaposed to parents’ progressive attitudes – suggest boys may not always view this reduced masculine pressure as beneficial. Moreover, boys’ conceptions of masculinity at home, though described as less restrictive, remained markedly traditional. Ostensibly prosocial paternalistic ideals regarding protecting and providing for female family members often carried a sexist, hierarchical undertone reminiscent of ambivalent sexism (Glick & Fiske, 1997). Accordingly, the “relaxed” character of home-based masculinity may not indicate an absence of constraint: Such norms, while less overtly punitive than school-based peer regulation, nonetheless prescribe narrow forms of masculinity and are closely intertwined with – and mutually reinforcing of – more dominance-oriented modes of gender regulation over time (Bareket et al., 2023).

### The influence of the manosphere

Our qualitative data include explicit endorsement of manosphere-aligned beliefs, with some boys directly affirming the legitimacy of dominance-oriented, misogynistic, and grievance-based accounts of masculinity. Boys often described proponents of the manosphere – such as Andrew Tate, David Goggins, and Jordan Peterson – as influential role models and benchmarks for masculinity. Even when these figures were not mentioned by name, their manosphere-style of masculinity extolling dominance, status, wealth, and misogyny was endorsed in a disquieting number of responses. Emblematically, some boys even framed endorsement of, and conformity to, traditional masculinity as a counter-culture rebellion against progressive ideologies that they suggest destabilize society by stripping men of their masculinity and thus their worth.

Although digital influence can be ironic, experimental and strategic, especially among youth (Boyd, 2014), the explicit manosphere endorsement in our sample does not support the idea that the manosphere narratives observed were merely rhetorical or ironic. At the same time, we note that endorsement during adolescence may reflect emergent, context-dependent meaning-making, rather than fully consolidated ideological commitment (Erikson, 1968). This distinction is theoretically important not because it questions the significance of manosphere endorsements, but because it highlights adolescence as a critical period during which manosphere narratives can become normalized, legitimized, and integrated into developing masculine identities.

The manosphere-like reaction against progressive ideals endorsed by Briarwood boys mirrors experimental evidence suggesting that backlash against non-traditional men intensifies when masculinity is perceived as threatened – especially among men who endorse traditional masculine norms to a greater degree (Iacoviello et al., 2021). Other recent experimental work, however, suggests such backlash is not immutable. Exposure to information framing men as becoming more feminine reduces men’s discomfort when imagining completing a feminine task, albeit only among those with less traditional attitudes about masculinity (Borinca et al., 2021). Similarly, exposure to narratives about gender-atypical sexual minorities weakens heterosexual men’s endorsement of traditional masculinity (Borinca & Gkinopoulos, 2025). Taken together, these studies suggest that traditional conceptions of masculinity we observe – as well as backlash against less traditional conceptions – are not inevitable or immutable, and may weaken as opportunities for, and archetypes of, less-restrictive masculinities emerge across development.

While we cannot generalize our results beyond Briarwood or across developmental time, the acceptance of manosphere narratives evident in these data is consistent with a broader societal shift in the social figures influencing boys’ attitudes more generally (see also McNulty & Birney, 2023; Wescott et al., 2024). In our data, boys extolled the value of adhering to traditional masculine norms, while describing those with less restrictive ideas about masculinity (such as parents and staff) as foolish, deluded, and contributing to societal decay. When they indicated knowledge of the controversy surrounding manosphere figures – or otherwise indicated that adults in their lives disapproved them – they explained away these controversies in order to justify their continued admiration. For example, one boy rationalized a prominent manosphere influencer’s imprisonment for sex trafficking as actually stemming from oppression of the influencer’s efforts to inspire boys and men, while another suggested the influencer had a “net-positive” effect due to his positive impact on men and boys. Taken together, these findings suggest that contemporary masculinity socialization may be increasingly organized around digital authority and peer-aligned grievance narratives, with important implications for how Australian institutions attempt to engage boys around gender, respect, and social responsibility.

### Limitations and future directions

Results of the current study provide indications that boys’ attitudes about masculinity stem from their peers, the broader school context, and online influences. However, our methods do not permit causal inferences regarding these links, and our results may stem from a range of potential sources not evident here. Likewise, the qualitative and single-sample nature of our analyses mean we cannot conclude whether our findings generalize to other boys or other contexts, particularly given that we predominantly sampled boys of European descent from relatively wealthy (but not elite) backgrounds. While young men from diverse socioeconomic and ethnic backgrounds do engage with masculinity influencers, manosphere engagement is disproportionately higher among those who are white, older, more highly educated, and living in higher-income households (Movember Institute of Men’s Health, 2025). Given that socioeconomic privilege may shape how dominance-oriented norms are legitimized and experienced, findings should be interpreted as reflecting masculinity socialization in the relatively affluent and European-descent contexts.

The written survey format may have shaped participants’ responses, potentially eliciting more stylized, normative, or socially performative accounts of masculinity rather than internalized attitudes. Though qualitative online surveys have many merits (e.g., efficient sampling of large populations, increased confidentiality; Braun et al., 2021), a deeper understanding of trends would have been possible using semi-structured interviews. Nevertheless, even idealized or performative responses are informative, as they reflect conceptions of masculinity perceived as socially desirable or legitimate within the school context. At the same time, the privacy and anonymity of written responses may mitigate social pressures – particularly salient in adolescence – that might bias face-to-face interviews or focus groups, where the presence of peers or interviewers may heighten impression-management concerns and constrain disclosure. In this sense, written surveys may afford boys greater latitude to reflect candidly on a socially policed topic, offsetting the more limited opportunities for follow-up questions.

Our results suggested that boys’ attitudes may be diverging from those of adults in their lives, but we highlight that two-thirds of the adults sampled were women. Divergences between these groups should thus be understood to be potentially biased towards boys’ differences to adult women, rather than adults in general. It is also worth noting that adolescence is a time of identity exploration (Erikson, 1968): some of the patterns we document may reflect normative adolescent processes of identity experimentation, rather than a strict generational ideological shift. Building on the strengths of the current research, future research could utilize a mixed-methods approach to explore the aims of this investigation with precision, specifically sampling an equal number of fathers/men to mothers/women. Such a study may like to track adolescents over time, disentangling generational differences from normal adolescent experimentation. This approach could further allow a clearer understanding of the relative extent to which men’s and women’s masculinity norms affect boys’ norms, as well as further investigate strategies to curb the influence of digital socialization forces.

## Conclusions

A qualitative analysis of 650 students, 287 parents, and 46 staff at an affluent independent faith-based boys’ school in metropolitan Australia suggest the continuing relevance of traditional masculinity to boys’ understandings of what it means to be men. Masculinity that was dominant, tough, status-driven, and independent was seen as sometimes regrettable and costly but nevertheless developmentally necessary. At the same time, masculinity was also context dependent, being more pro-social at home and dominance-oriented at school. Many boys’ masculine attitudes were reinforced by and reflected manosphere narratives and counter-culture resistance to progressive ideology. These findings, as well as a substantial divergence between boys’ views and those of parents and school staff, suggest that adult influence on boys’ masculinity attitudes is increasingly eclipsed by peer influence and louder, digitally mediated sources of authority.

## Data Availability

The qualitative nature of the data precludes data sharing to protect participant confidentiality.

## Appendix A

### AS1. Qualitative questions

*To capture a detailed and broad range of responses, nine qualitative survey questions were developed by the research team for this study and independently approved by an advisory group within Briarwood College. All participants were asked these questions, with the exception of QP6, which was asked only to parents and staff. All questions were free responses, with participants allowed to write as much or as little as they wanted.*

Q1. What is masculinity and what characteristics do you associate with it? [open text]

Q2. What does masculinity look like, feel like or sound like at Briarwood College? [open text]

Q3. What does masculinity look like, feel like or sound like in your home? [open text]

Q4. Should definitions of masculinity be the same for everyone? Why or why not? [open text]

Q5. How does your view of masculinity influence you (impact the choices you make)…? [open text]

Q5.1. at school/work

Q5.2. at home

Q5.3. in the community

QP6. How does your view of masculinity influence you (impact the choices you make) as a parent? [open text]

Q7. Is your parents’ definition of masculinity different from your own? [discrete choices]

- No, not different at all
- Yes, somewhat different
- Yes, very different

Q7.1. [if “b” or “c” are selected] How is it different? [open text]

Q8. In your opinion, what does the ideal man do or say: [open text]

Q8.1. in the classroom

Q8.2. on the sporting field

Q8.3. at home

Q8.4. in the community

Q8.5. in the school yard

Q9. I wish masculinity at Briarwood College looked like, felt like, or sounded like… [open text]
